# Different effect of chemical refining process on Baneh (*Pistacia atlantica var mutica*) kernel oil: Regeneration of tocopherols

**DOI:** 10.1002/fsn3.2515

**Published:** 2021-08-04

**Authors:** Aniseh Zarei Jelyani, Javad Tavakoli, Hannan Lashkari, Mahmoud Aminlari

**Affiliations:** ^1^ Food Science and Technology Department Sarvestan Branch Islamic Azad University Sarvestan Iran; ^2^ Department of Food Science and Technology Faculty of Agriculture Jahrom University Jahrom Iran; ^3^ Department of Food Science and Technology Zarin Dasht Branch Islamic Azad University Zarin Dasht Iran; ^4^ Department of Biochemistry School of Veterinary Medicine Shiraz University Shiraz Iran

**Keywords:** antioxidant activity, antioxidant compounds, chemical properties, qualitative parameters

## Abstract

The present study was conducted to investigate the impact of refining process on the chemical properties (fatty acid composition and tocopherols, sterols, and polyphenolic contents), qualitative parameters (peroxide value, acid value, and p‐anisidine value), and antioxidant activity (DPPH radical scavenging assay and FRAP test) of Baneh (*Pistacia atlantica* var *mutica*) kernel oil. The results revealed that the refining process had no significant effect on the fatty acid composition. A major finding of this research was the increase in the tocopherol and sterol content up to the bleaching stage followed by their decrease in the deodorizing phase. Some tocopherol and sterol compounds in crude oil were dimerized or attached to other compounds by ester bonding, which are released during some stages of the refining process and this factor is responsible for their increase. In fact, during this process, these compounds are regenerated. The occurrence of this phenomenon in the refining process improved the DPPH radical scavenging power of Baneh kernel oil up to the bleaching stage. Moreover, the content of phenolic compounds decreased after refining of Baneh kernel oil, and only in the deodorizing stage, an increase of these compounds was observed. In general, the results of this study showed that the refining process had a completely different effect on the antioxidant compounds (especially tocopherols) compared to other oils.

## Graphical abstract

In this research, for the first time in the world, Baneh (Pistacia atlantica) kernel oil was refined. Various researches have been done about this oil in its crude form, and it shows the high antioxidant activity and oxidative stability of this oil. Consumption of this oil is expanding, and this research provides useful information.

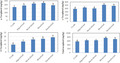

## 1 INTRODUCTION

One of the eleven common species of pistachio is Baneh (*Pistacia atlantica*), which grows wildly in different parts of Iran. The area under the cultivation of these trees in Iran is more than 1.2 million hectares with approximately 100 million trees (Hatamnia et al., 2014; Moslehi et al., 2021). The annual fruit yield of this tree is 30–40 kg. The fruit of the Baneh tree consists of three parts: the outer oily green hull, the wood hull, and the kernel. Baneh kernels make up to 25% of Baneh fruit, and its oil yield is close to 50% (Padulosi & Hadj‐Hassan, 1998; Tavakolli et al., 2019b). This species has three varieties, with mutica being the most famous and common Baneh variety, which comprises 90% of the population of these trees in Iran (Tavakoli et al., 2019b). There have been various researches on Baneh crude oil. Farhoosh et al. (2008), for example, proved that Baneh crude oil has a very high oxidative stability at 170°C (Farhoosh et al., 2008). Tavakoli et al. (2019) investigated the antioxidant activity of crude kernel oil. The results of oven test, free radical scavenging DPPH assay, reducing power of Fe^+2^ (FRAP test), and rancimat showed that Baneh crude oil and its unsaponifiable matters had higher antioxidant activity compared to rice bran and sesame oils, known as oils with high oxidative stability (Tavakoli et al., 2019a). Also in two different studies, it was proved that Baneh kernel oil and its unsaponifiable matters in the amounts of 0.1% and 100 ppm had antioxidant power equal to TBHQ (the most common and powerful synthetic antioxidant in the food industry), respectively, during 48 hr of frying canola oil (Sharayei et al., 2011a,2011b). Therefore, previous research has shown that Baneh crude oil, with 85% of unsaturated fatty acids, is an oil with considerable oxidative stability and antioxidant activity. The majority of previous studies on Baneh kernel oil have focused on its crude oil, and there is no study on refined Baneh kernel oil.

Unpleasant compounds such as free fatty acids, phospholipids, and oxidation compounds have a negative effect on the physicochemical properties, sensory properties, and shelf life of edible oils (Chang et al., 2016; Mahjoob et al., 2017; Pestana et al., 2008; Zhu et al., 2016). During the process of chemical or physical refining, these compounds are removed and increase the quality of edible oils (Liu et al., 2019; Pan, Li, et al., 2020). In general, chemical refining includes degumming, neutralizing, bleaching, and deodorizing. Although the refining process is necessary to maintain the quality of edible oils, this process can nutritionally eliminate beneficial oil compounds (Kreps et al., 2014; Pan, Wen, et al., 2020). For this reason, various studies have been conducted on the effect of the refining process on the beneficial and nutritious compounds of edible oils. For example, Liu et al. (2019) investigated the effect of the refining process on the chemical structure of rice bran oil. The results showed that the fatty acid and tocopherol components of this oil did not change significantly compared to crude oil, although the amount of other unsaponifiable compounds was significantly decreased.

In another study, Pan, Li, et al. (2020) reported that the chemical refining process of perilla seed oil eliminated tocopherols by 5%. The refining process has not always been accompanied by a slight reduction in useful compounds. In a study on the effect of the refining process on rapeseed oil micronutrients, Wu et al. (2019) reported that the levels of tocopherols, sterols and polyphenols were reduced by 88%, 35% and 75%, respectively. In general, the refining process should be done in such a way that the maximum of unsuitable compounds and the minimum of useful compounds are removed. Therefore, considering that Baneh crude oil is known as an oil with high oxidative stability which needs to be refined for general consumption, and so far no research has been done on the refining process of this oil, the present study was carried out to investigate the impact of chemical refining process on the chemical composition, quality parameters, and antioxidant activity of Baneh kernel oil.

## 2 MATERIALS AND METHODS

### 2.1 Materials

The fruit of Baneh tree was collected from the forests of Meymand City in Fars Province (autumn 2019) and kept at 4°C before oil extraction. All standards, chemicals, and solvents were provided by Sigma and Merck.

### 2.2 Oil extraction

Baneh fruit was first dried in shade at ambient temperature. Then, fruits were peeled and the kernel was taken for oil extraction. In the next step, Baneh kernel oil was extracted by cold pressing at 25°C. The suspended solids were allowed to precipitate from extracted oil for one week. Finally, the oil was filtered and stored in dark containers containing nitrogen at 4°C (Ozcan et al., 2019).

### 2.3 Chemical Refining

In the degumming process, the crude oil of Baneh kernels was heated to 70°C and then 0.2% phosphoric acid was added with slow agitation for 30 min. Then, it was washed three times with water. The oil settled for 30 min to precipitate the suspended solids. In the neutralization stage, the oil temperature reached 80°C and the neutralization was performed by adding 3 N sodium hydroxide solution. For bleaching process, 1% bleaching earth (w/w) was added to the neutralized oil with vigorous stirring. At this stage, heating at 110°C was done for 30 min. In the dewaxing process, the bleached oil was cooled gradually at 15°C for 4 hr for crystallization of wax and filtered to remove the wax. To deodorize the oil, the bleached oil was vacuumed at 250°C and then cooled to 45°C (Pal et al., 2015).

### 2.4 Fatty acid composition

The fatty acid composition of the vegetable oils was determined by gas–liquid chromatography according to the method previously established (Tavakoli, Emadi, et al., 2018). Gas chromatography HP‐5890 (Agilent) equipped with a CP 88 3,400 (Varian) capillary column of fused silica (120 m in length × 0.25 mm in internal diameter, 0.25 μm film thickness) using a flame ionization detector (FID) was used. The carrier gas was helium with a flow rate of 0.8 ml/min.

### 2.5 Calculated oxidizability value and iodine value

The calculated oxidizability (Cox) value for the oil samples was determined according to the proposed equation by Fatemi and Hammond (1980):

Cox = [1(18:1% + 10.3(18:2%) + 21.6(18:3%)]/100.

Iodine value (IV) was measured based on the AOAC Official Methods 920.158 (Hanus method) (AOAC, 2005).

### 2.6 Acid value, peroxide value, and p‐anisidine value

The determination of acid value (AV), peroxide value (PV), and p‐anisidine value (p‐AV) of the different oil samples was done based on the method ascribed by Asadi & Farahmandfar, 2020, Roshanpour et al. (2021), and Estakhr et al. (2020), respectively.

### 2.7 Total phenolic content

Total phenolic content was measured as established by Tavakoli et al. (2019b). Folin–Ciocalteu reagent was used in this spectroscopic method at 765 nm (model 160A, Shimadzu, Tokyo) and calculated by a calibration curve (*R^2^* = 0.99) performed with gallic acid (0 to 0.4 mg/ml).

### 2.8 Determination of DPPH radical scavenging activity and FRAP test

Evaluation of DPPH radical scavenging assay was done using method described by Farahmandfar et al. (2019), Farahmandfar et al. (2018), and Farahmandfar and Ramezanizadeh (2018). Also, the method of Tavakoli et al. (2019a) was used for ferric reducing antioxidant power (FRAP) test.

### 2.9 Analysis for sterol compounds

To quantify sterol compounds of oil samples, a gas chromatography system (HP‐5890; Agilent) equipped with a SE 54 CB column (Macherey‐Nagel, Duren, Germany; 50 m long, 0.25 mm ID, 0.25 μm film thickness) was used. The parameters used can be summarized as follows: The carrier gas used was helium with a flow rate of 0.8 ml/min. The oven temperature gradient was raised 5°C from 160 C to 200 C for each 5 min; temperatures of the injector and the detector were adjusted as 210 C and 300 C, respectively (Tavakoli, Hashemi, et al., 2018).

### 2.10 Analysis for tocopherol compounds

Tocopherol compounds in oil samples were determined using a high‐performance liquid chromatography (HPLC) system (Waters ACQUITY UPLC^®^ System) with a Spherisorb column (25 cm × 4 mm i.d., WATERS) packed with silica (5 μm particle size), and a fluorescence detector operating at an excitation wavelength of 290 nm and an emission wavelength of 330 nm was utilized. The used mobile phase consisted of acetonitrile and water (90:10, v/v) at a flow rate of 0.5 ml/min. Tocopherols in the test samples were verified by the comparison of the retention time with the available reference standards (Tavakoli, Hashemi, et al. (2018)).

### 2.11 Statistical analysis

All experiments were conducted in three replications, and the obtained results were analyzed by the aid of analysis of variance (ANOVA) (MStatC). Moreover, the Slide Write and Excel software were employed to design regression and graphs, respectively. Meanwhile, the Duncan's test was applied to compare the mean values.

## 3 RESULTS AND DISCUSSION

### 3.1 Fatty acid composition, cox value, and iodine value

The effect of the refining process on the fatty acid profile, Cox value, and iodine value of Baneh kernel oil is presented in Table 1. No significant difference was observed in the amount of various fatty acids. In a study, Liu et al. (2019) investigated the effect of the refining process on the chemical composition of rice bran oil. Their results showed that the refining process has no significant effect on the fatty acid composition of rice bran oil. Pal et al. (2015) also reported that the refining process had no significant effect on the fatty acid composition of sunflower oil. Similar results were reported in another study on evening primrose oil (Pan, Li, et al., 2020) that was consistent with the results of the present study.

**TABLE 1:** Effect of refining process on fatty acid structure, oxidizability (Cox), value and iodine value of Baneh kernel oil

Fatty acid (%)	Crude	Degumming	Neutralization	Bleaching	Deodorization
C14:0	0.08 ± 0.02 a	0.09 ± 0.03 a	0.09 ± 0.02 a	0.08 ± 0.03 a	0.09 ± 0.02 a
C16:0	10.01 ± 0.3 b	10.25 ± 0.22 ab	10.52 ± 0.32 ab	10.23 ± 0.21 ab	10.76 ± 0.3 a
C16:1	1.12 ± 0.07 a	1.17 ± 0.05 a	1.15 ± 0.04 a	1.32 ± 0.03 a	1.22 ± 0.05 a
C18:0	2.15 ± 0.12 a	2.12 ± 0.11 a	2.05 ± 0.08 a	2.23 ± 0.10 a	2.15 ± 0.07 a
C18:1 t	0	0	0	0	0
C18:1	52.5 ± 0.42 a	52.09 ± 0.34 a	51.93 ± 0.41 a	52.09 ± 0.32 a	51.95 ± 0.37 a
C18:2 t	0	0	0	0	0.04 ± 0.02
C18:2	33.2 ± 0.24 a	33.1 ± 0.27 a	33.1 ± 0.31 a	33.11 ± 0.22 a	32.72 ± 0.29 a
C18:3ῳ3	0.42 ± 0.05 a	0.46 ± 0.04 a	0.45 ± 0.03 a	0.46 ± 0.02 a	0.45 ± 0.03 a
C18:3ῳ6	0.3 ± 0.03 a	0.31 ± 0.04 a	0.31 ± 0.05 a	0.33 ± 0.04 a	0.29 ± 0.04 a
C20:1	0.12 ± 0.04 a	0.15 ± 0.02 a	0.14 ± 0.03 a	0.16 ± 0.04 a	0.14 ± 0.03 a
Oxidizability (Cox) value	4.1 ± 0.12 a	4.12 ± 0.08 a	4.1 ± 0.07 a	4.1 ± 0.06 a	4.07 ± 0.08 a
IV (g of I_2_ /100 g oil)	104.4 ± 1.1 a	104.1 ± 0.9 a	104 ± 1.2 a	104.2 ± 1.2 a	103.3 ± 1 a

Note

Mean ± *SD* within a row with the same uppercase letters is not significantly different at *p* < .05.

Mirrezaie et al. (2016) reported that the effect of the refining process on olive oil caused significant changes in the fatty acid composition of olive oil. Differences in fatty acid results can be attributed to different methods of olive oil refining and the oil studied in the present study. Examination of the fatty acid composition of crude and refined Baneh kernel oil also showed that oleic acid (51.93–52.5%) was the predominant fatty acid, followed by linoleic acid (33.1–33.72%) and palmitic acid (10.01–10.76%), respectively, which was close to the results reported by Farhoosh et al. (2008). The absence of trans fatty acids in Baneh kernel oil during the refining process was one of the interesting results of this study. In investigating the effect of the refining process on rapeseed oil, Wu et al. (2019) reported that the amount of trans fatty acids increased by up to 5% during the deodorizing phase. Another study found that the content of trans fatty acids in rice bran oil increased during the refining process but was less than 1% (Van Hoed et al., 2006). Moreover, changes in Cox value and iodine value showed that there was no significant difference in these factors during refining of Baneh kernel oil. Cox value and iodine value are directly related to the unsaturation of fatty acids. Due to the fact that there was no significant difference in the amount of unsaturated fatty acids during the refining process, these factors did not change.

### 3.2 Qualitative parameters (AV, PV, and p‐AV)

**FIGURE 1:**
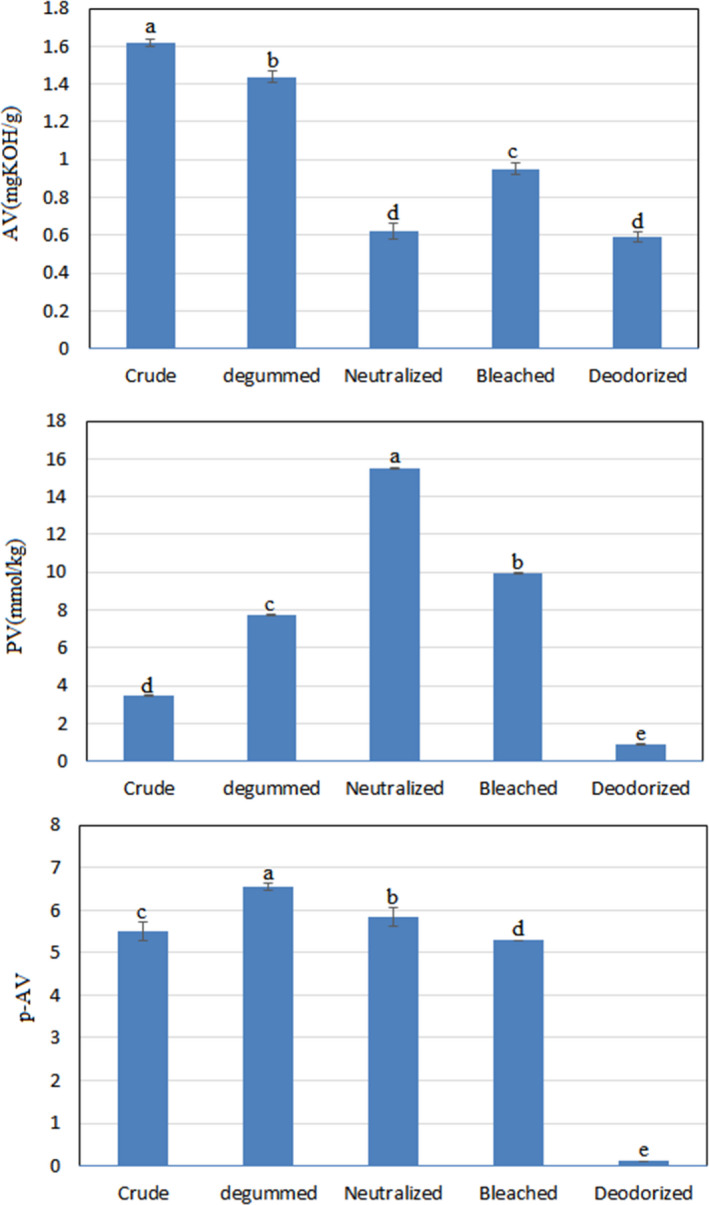
Effect of refining process on acid value (AV), peroxide value (PV), and p‐anisidine value (p‐AV) of Baneh kernel oil. Mean ± *SD* within a column with the same uppercase letters is not significantly different at *p* < .05

The effect of the refining process on AV, PV, and p‐AV is shown in Figure 1. The AV levels of crude, degummed, neutralized, bleached, and deodorized oils of Baneh kernel were 1.61, 1.44, 0.62, 0.94, and 0.59, respectively. The greatest reduction of this parameter was observed in the neutralization stage, followed by the deodorization and degumming stages, respectively. In contrast, the bleaching phase increased the AV compared with the neutralization phase. In the neutralization phase, saponification of free fatty acids with NaOH reduced the AV (Dumont & Narine, 2010; Liu, 2013). Moreover, in the deodorizing phase, due to the application of vacuum and the use of water vapor, some fatty acids had suitable kinetic energy and separated from the oil (Zhu et al., 2016). According to Codex Stan 210–1999 Codex Standard For Named Vegetable Oils, the AV content of edible oils should be less than 0.6 (Liu et al., 2019). Due to the fact that the AV level of Baneh kernel oil decreased to 0.59, it indicated the appropriate effect of the refining process on free fatty acids.

Pal et al. (2015) examined the effect of the refining process on the quality of sunflower oil and showed that the AV content of crude oil decreased from 1.1 to 0.24 in laboratory refined oil and 0.13 in commercial refined oil. The reason for the difference in the results of this study and the present study can be attributed to the difference in the initial amount of AV in Baneh kernel and sunflower oils, as well as the differences in the refining method. In another study, Liu et al. (2019) reported that the amount of AV in the rice bran oil refining process was reduced. Only degumming phase increased AV and bleaching stage also had a positive effect on AV of rice bran oil, which was different from the results of the present study. In accordance with the results of the present study, Pan, Wen, et al. (2020) a) and Zhu et al. (2016) reported that the AV of evening primrose oil and peanut oil decreased during the refining process, but the bleaching step increased their AV.

The PV changes of Baneh kernel oil during the refining process are shown in Figure 1. The PV levels of crude, degummed, neutralized, bleached, and deodorized oils of Baneh kernel were determined to be 3.46, 7.7, 15.5, 9.9, and 0.9 mmol/kg, respectively, with a significant difference between them. As seen, an increasing trend was observed in the gumming and neutralization due to the presence of air, heat, humidity, and light during these stages and the increase of the formation of the initial oxidation compounds (Zhu et al., 2016).

In the present study, during the bleaching and deodorization stages, the amount of PV decreased by 36 and 90.9%, respectively, which was consistent with the research of Zhu et al. (2016) on peanut oil and Farhoosh et al. (2009) on soybean oil, but in some other studies, the trend of PV changes of different oils during the refining process was completely reduced (Pan, Li, et al., 2020; Pestana et al., 2008). According to Codex Stan 210–1999 Codex Standard For Named Vegetable Oils Standard, the PV level of edible oils should be less than 10.0 mmol/kg (Liu et al., 2019), which according to Table 1 showed that the final refined oil had much less PV than this amount, showing suitable conditions.

Examination of p‐AV changes during the refining process showed that in the degumming stage, the value of this index increased from 5.5 to 6.55 and in the next stages, it decreased, so that p‐AV in neutralized, decolorized, and deodorized oils were 5.83, 5.29, and 0.11 respectively (Figure 1). A noteworthy point was the very significant effect of the deodorizing stage on the reduction (98%) of this index compared to the previous stage. The reason for this result can be attributed to the escape of aldehyde compounds and the creation of a vacuum in the deodorizing stage. Zhu et al. (2016) reported that during the refining process of peanut oil, p‐AV increased to the bleaching stage and then in the deodorizing stage, the amount of these compounds decreased. Pan, Li, et al. (2020) reported different results in another study. The amount of p‐AV during the evening primrose oil refining process was increasing. This result was attributed to the breakdown of hydroperoxides and the formation of oxidation secondary compounds. Wu et al. (2019) examined the effect of the refining process on rapeseed oil and showed that in the degumming and deodorizing stages, the p‐AV level decreased compared to their other stage.

### 3.3 Tocopherol content

**FIGURE 2:**
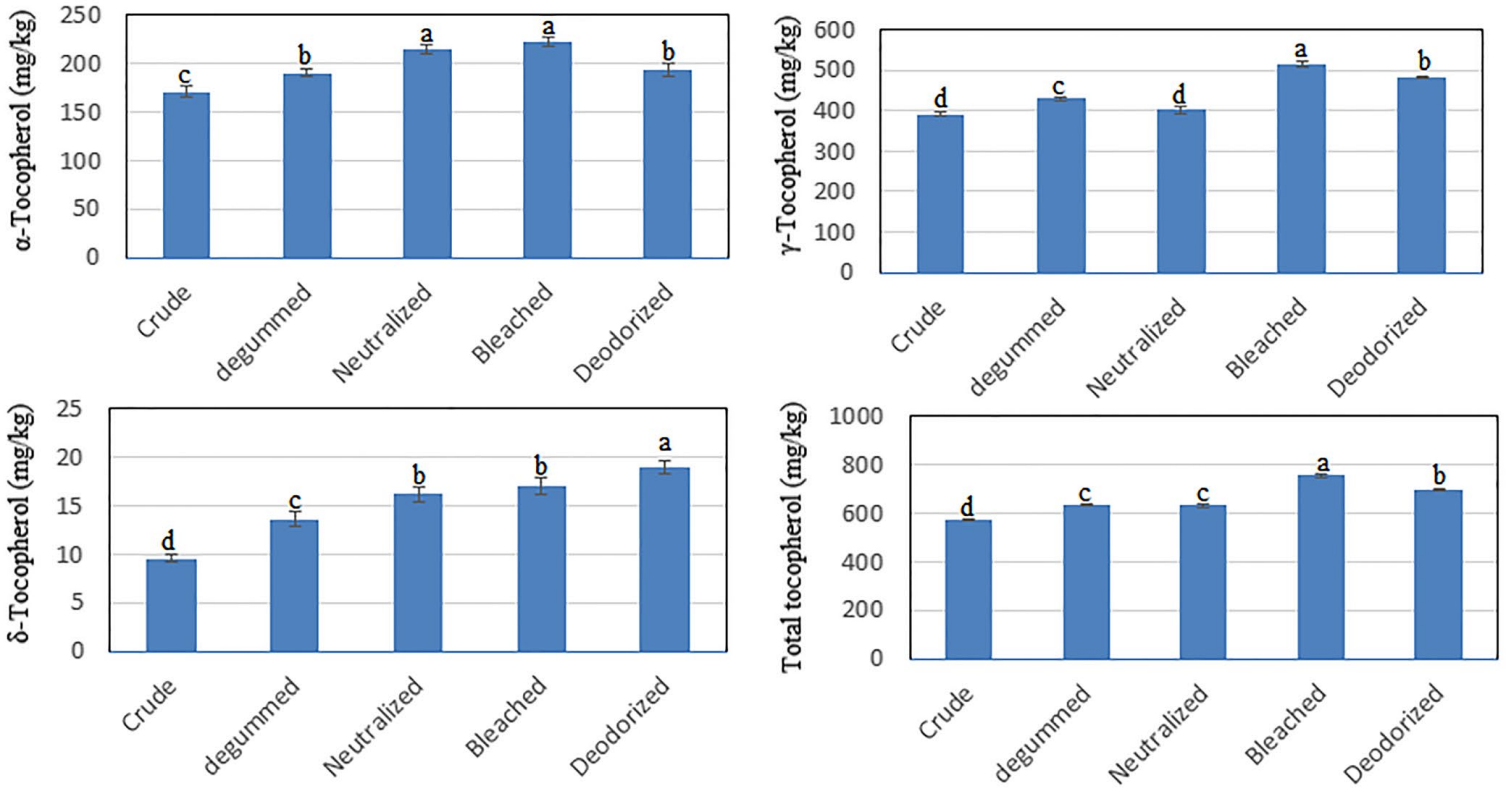
Effect of refining process on tocopherol composition of Baneh kernel oil. Mean ± *SD* within a column with the same uppercase letters is not significantly different at *p* < .05

Changes in tocopherol content during the Baneh kernel oil refining process are shown in Figure 2. As seen, the amount of total tocopherol content in crude, degummed, neutralized, bleached, and deodorized oils of Baneh kernel were determined to be 572, 634, 633, 754, and 694 mg/kg, respectively. The results showed that the amount of tocopherol increased up to the bleaching stage (32%) and only in the deodorizing stage, a decrease of these compounds was observed. The trend of changes in these compounds in the present study was different from the results of most other studies that reported that the amount of tocopherol content decreases during the refining process (Zhu et al., 2016; Wu et al., 2019; Vanessa Ribeiro et al., 2012; Gotor & Rhazi, 2016; Tasan & Demirci, 2005), but in some studies, similar results were found. Liu et al. (2019) and Pestana et al. (2008) reported in two different studies that during the refining process, the amount of tocopherol compounds in rice bran oil increased up to the bleaching stage and then in the deodorizing stage, a decrease was observed. Chew et al. (2016) also reported that the trend of changes in tocopherol compounds in the refining process of kenaf seed oil was not regular. An increase in these compounds was observed in the degumming and bleaching stages and a decrease in the neutralization and deodorizing stages. The reason for the increase in tocopherol compounds was attributed to their regeneration by breaking the dimeric bonds between tocopherols or breaking the ester bond of tocopherols and other compounds (Chew et al., 2016; Liu et al., 2019; Pestana et al., 2008).

Similar results were reported by Rossi et al. (2001) on the regeneration of tocopherol compounds. Regarding the reason for the increase in tocopherol compounds in the present study, it can be said that tocopherols are not always free. These compounds can form a matrix with other compounds. It is also possible to bind tocopherol compounds to each other and form dimers. Baneh kernel oil seems to contain nonfree tocopherol compounds that are released after refining to the bleaching stage and the amount of these compounds increases. Also in the deodorizing stage, due to the thermal degradation of tocopherol compounds at high temperatures that occur during oxidation reactions or other chemical reactions, a decrease in these compounds was observed (Liu et al., 2019; Verleyen and Et al. 2002, Tasa & Demirci, 2005).

Examination of the tocopherol structure of Baneh kernel oil also showed that gamma tocopherol was the major tocopherol compound followed by alpha tocopherol (Figure 2). Sigma tocopherol was also measured in small amounts (<20 ppm). The trend of changes in tocopherol compounds during the refining process showed that in the fully refined Baneh kernel oil, more regeneration of gamma tocopherol (23.3%) was observed than alpha tocopherol (12.9%). Pestana et al. (2008) reported that among the different isomers of tocopherol, only the amount of alpha tocopherol increased during the purification process and other isomers followed a decreasing trend. Zhu et al. (2016) also investigated the effect of peanut oil refining process on tocopherol compounds and concluded that at the end of the refining process, among the tocopherol isomers, alpha‐ and gamma‐tocotrienol increased compared to crude oil and the rest of the isomers had a decreasing trend.

### 3.4 Sterol compounds

The effect of the refining process on the sterol structure of Baneh kernel oil is given in Table 2. As seen, the content of total sterol compounds in the oil at the end of the refining process was not statistically significant different with crude oil. It was also found that the amount of these compounds increased in the neutralization and decolorization stage and decreased in other stages. According to many previous studies, the amount of sterol compounds is expected to decrease during the refining process of edible oils (Ghazani et al., 2013; Gotor & Rhazi, 2016; Pan, Li, et al., 2020; Wu et al., 2019). However, an increase in sterol compounds during the purification process was also observed in some other studies. Liu et al. (2019) reported in a study that the level of sterol compounds in rice bran oil increased during the degumming stage and decreased during other refining stages. Also, in another study, it was reported that degumming increased the total sterol composition of soybean oil and palm oil relative to its crude state. The levels of sitosterol and campesterol in soybean oil and the amount of campesterol in palm oil increased (Verleyen et al., 2002). Examination of the sterol compounds of Baneh kernel oil showed that β‐sitosterol (49.5%–54.6%) had the highest amount of these compounds, followed by campesterol (36.1%–40.1%), brassicasterol (8.8%–9.8%), and stigmasterol (0.3%–0.6%). Also, among the sterol compounds, increase in β‐sitosterol and campesterol was observed during the purification process, which was consistent with the study of Verleyen et al. (2002) on soybean oil. In justifying the increase in sterol compounds, it can be said that these compounds have the ability to bind to other compounds and form a matrix. During the neutralization and decolorization stage, these compounds are released and we are faced with increase of sterols. In the deodorizing stage, due to the use of high temperatures, these compounds underwent thermal decomposition and their amount was reduced.

**TABLE 2:** Effect of refining process on the phytosterol composition of Baneh kernel oil

Phytosterol composition	Crude	Degumming	Neutralization	Bleaching	Deodorization
Brassicasterol	67 ± 3a	74 ± 4a	73 ± 3a	70.1 ± 2a	68.01 ± 3a
Campesterol	292 ± 5bc	283 ± 6c	314 ± 6a	301 ± 5b	275 ± 7d
stigmasterol	4.1 ± 0.2a	4.5 ± 0.4a	3 ± 0.2b	2.7 ± 0.2b	2.8 ± 0.2b
sitosterol	402 ± 6b	394 ± 7bc	383 ± 8c	422 ± 8a	415 ± 6a
Total	766 ± 8bc	756 ± 7c	773 ± 8b	796 ± 9a	761 ± 5c

Note

Mean ± within a row with the same uppercase letters is not significantly different at *p* < .05.

### 3.5 Phenolic compounds

**FIGURE 3:**
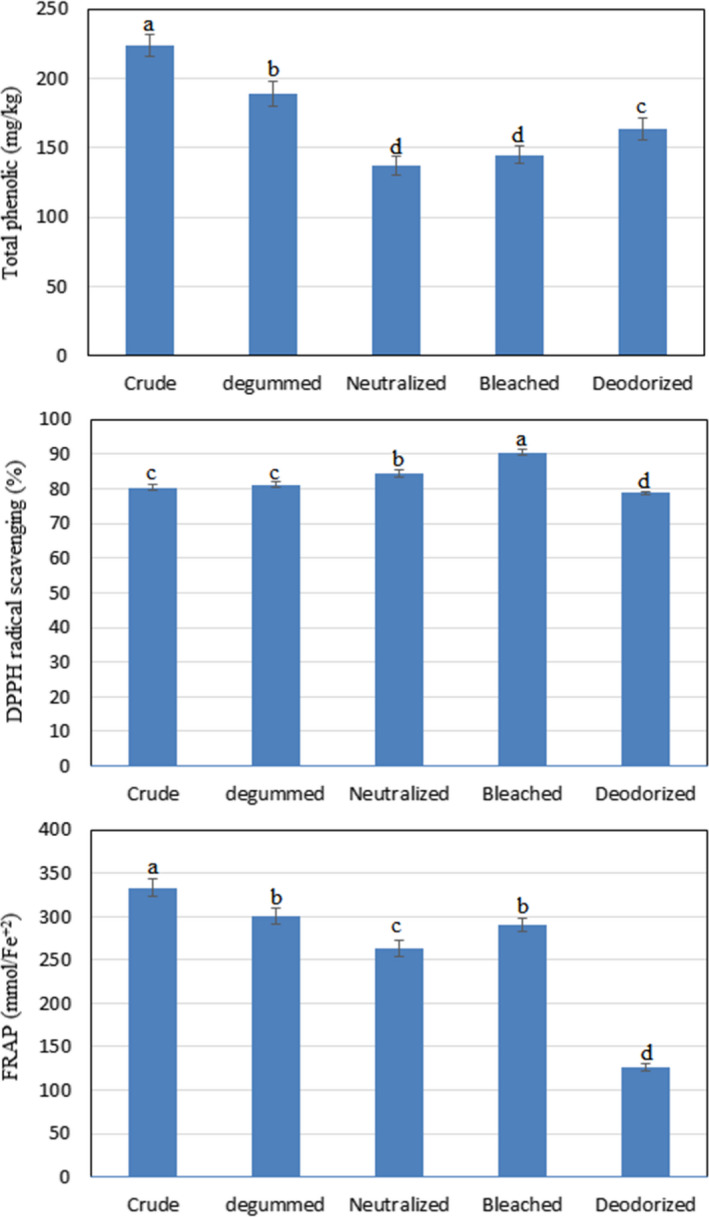
Effect of refining process on FRAP test, DPPH radical scavenging assay, and total phenolic of Baneh kernel oil. Mean ± *SD* within a column with the same uppercase letters is not significantly different at *p* < .05

The effect of the refining process on the phenolic compounds of Baneh kernel oil is shown in Figure 3. The phenolic compounds of crude, degummed, neutralized, bleached, and deodorized oils of Baneh kernel were determined to be 224, 189, 137, 145, and 163 (mg/kg), respectively. As seen, the amount of these compounds decreased during degumming and neutralization and remained fixed during bleaching and increased during deodorization. In most previous research, it has been reported that the amount of phenolic compounds in edible oils decreases during the refining process (Ghazani et al., 2013; Pan, Wen, et al., 2020; Wu et al., 2019). However, studies have also shown that in some stages of the oil refining process, the amount of phenolic compounds increased (Liu et al., 2019). Phenolic compounds structurally include a wide range of compounds from simple molecules to complex polymers. These compounds also exist in two forms, free and bonded with other compounds (Pérez‐Jiménez & Torres, 2011).

The increase in these compounds may be due to the fact that during the deodorizing phase, phenolic compounds bonded with other compounds may be released and the amount of these compounds may increase. Complex phenolic compounds may also be broken down by temperature, especially during the high‐temperature deodorizing phase, and converted into several simpler phenolic compounds, resulting in an increase in the amount of these compounds. Mirrezaie Roodaki et al. (2016) in a study investigated the effect of thermal process on the properties of olive oil. The results showed that the phenolic compounds of virgin and refined olive oil increased by 39% and 42%, respectively, after one hour of thermal processing at 180°C. Due to the fact that in the present study, a temperature of 250°C was used in the deodorizing phase, and it is possible to increase the phenolic compounds similar to the research of Mirrezaie Roodaki et al. Also, during the Baneh kernel oil refining process, the greatest reduction of these compounds was neutralized. Polyphenols are polar compounds and are usually weak acids that are easily removed from the oils with aqueous solutions, especially when neutralized with sodium hydroxide (Szydłowska‐Czerniak et al., 2008; Liu et al., 2019).

In another study, it was reported that during the refining process, the amount of phenolic compounds in perilla seed oil in the deodorizing stage was significantly increased compared to the bleaching stage, which was consistent with the results of the present study. Pan, Wen, et al. (2020).

### 3.6 Antioxidant activity

The impact of Baneh kernel oil refining process on its antioxidant activity is shown in Figure 3. As seen, the rate of DPPH radical scavenging of crude, degummed, neutralized, bleached, and deodorized Baneh kernel oils was 80.3%, 81.2%, 84.2%, 90.5%, and 78.7%, respectively. The trend of free radical scavenging changes was ascending until the bleaching stage, where the greatest increase was observed in the bleaching stage and then decreased in the deodorizing stage. Many previous studies have confirmed that the free radical scavenging power of edible oils decreases during the refining process, which is related to the reduction of antioxidant compounds during the process (Liu et al., 2019; Pan, Li, et al., 2020; Szydłowska‐Czerniak et al., 2008).

**FIGURE 4:**
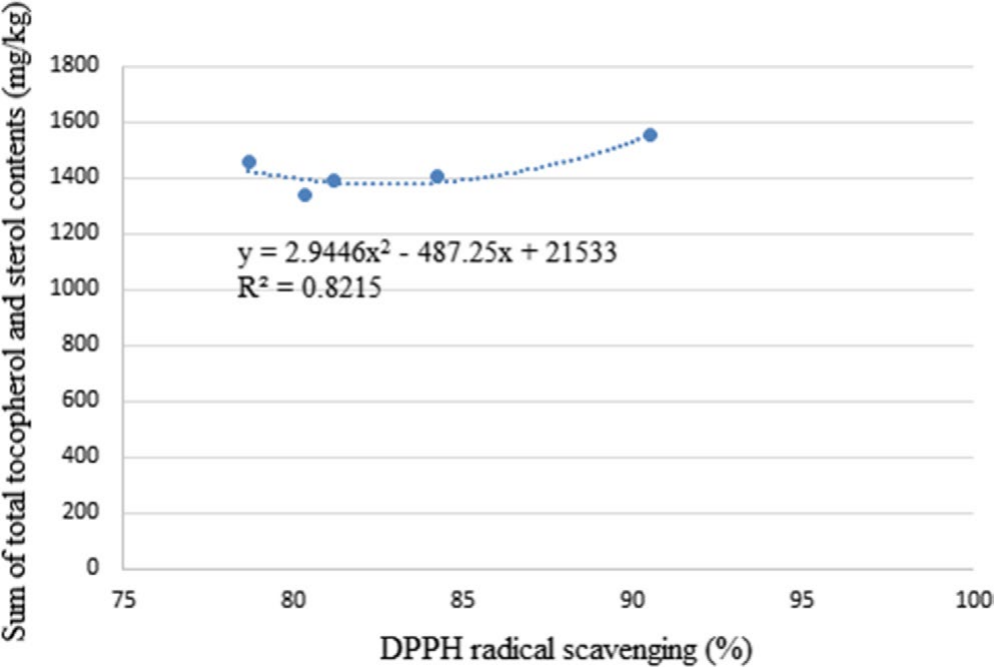
Correlation between sum of total tocopherol and sterol contents and DPPH radical scavenging power of Baneh kernel oil

Furthermore, in some other studies, an increase in free radical scavenging power was observed in some refining stages. Chew et al. (2016) reported that the free radical scavenging power of kenaf oil increased by 84% during the refining process due to the increase in tocopherol compounds of this oil. Szydłowska‐Czerniak et al. (2011) investigated the effect of the refining process on the antioxidant capacity of palm oil. The results showed that the amount of DPPH radical scavenging during the refining process first increased and then decreased, which was consistent with changes in phenolic compounds. In another study, Pan, Wen, et al. (2020) reported that the amount of free radical scavenging of perilla seed oil decreased and then increased during the refining process. In the present study, the changes in DPPH radical scavenging during the Baneh kernel oil refining process were consistent with changes in the sum of total tocopherol compounds and total sterol compounds (Figure 4). The amount of tocopherol and sterol compounds increased until the bleaching stage and decreased in the deodorizing stage, which was similar to the changes of DPPH radical scavenging.

How the FRAP test changes is also shown in Figure 3. The Fe^+2^reduction power of crude, degummed, neutralized, bleached, and deodorized Baneh kernel oils was determined to be 333, 301, 263, 290, and 126 mmol Fe^+2^/L, respectively. As can be seen, after the end of the refining process, the reducing power of Baneh kernel oil was reduced by 62% compared to crude oil. Moreover, in the bleaching stage, the amount of this factor increased by 10% compared to the neutralization stage. Unlike the DPPH radical scavenging test, the modifications of the FRAP test were not consistent with the changes in tocopherol and sterol compounds, while up to the bleaching stage were somewhat consistent with the results of changes in phenolic compounds. This difference is attributed to the differences in the mechanism of DPPH and FRAP tests. In some previous studies, it has been confirmed that during the refining process, the amount of iron reducing power is not completely reduced, and in some stages, an increase in the value of this factor was observed. The results of Pan, Wen, et al. (2020) showed that during the perilla seed oil refining process, the reducing power of Fe^+2^ increased in the degumming, neutralizing, and deodorizing stages and decreased during the bleaching stage. Moreover, the changes in the FRAP test were not consist to the changes in the antioxidant composition of perilla seed oil during the refining process. In another study, Szydłowska‐Czerniak and Łaszewska (2017) reported that the iron reducing power of rapeseed oil increased 1.3 times compared to crude oil during the degumming process.

## 4 CONCLUSIONS

The results of present study indicated that the trend of change in antioxidant compounds was different with other edible oils. The amount of tocopherol and sterol compounds increased during the refining process up to the bleaching stage and then decreased during the deodorizing phase. The increase in these compounds was attributed to their regeneration. Some of these compounds are dimerized or esterified with other compounds that are released during degumming, neutralization, and bleaching, and as a result, the amount of these compounds increases. Moreover, the content of phenolic compounds decreased after refining of Baneh kernel oil and only in the deodorizing stage, an increase of these compounds was observed. Overall, our results showed that the chemical refining process had a positive effect on the properties of Baneh kernel oil. Finally, other studies such as optimizing the Baneh kernel oil refining process or examining the effect of other refining methods on the properties of Baneh kernel oil can be suggested.

## CONFLICTS OF INTEREST

The authors declare no conflict of interest.

## AUTHOR CONTRIBUTIONS

**Aniseh Zarei:** Conceptualization (equal); Data curation (equal); Investigation (equal). **javad tavakoli:** Formal analysis (equal); Funding acquisition (equal); Investigation (equal); Methodology (equal); Software (equal); Supervision (equal); Validation (equal); Writing‐original draft (equal). **Hannan Lashkari:** Methodology (equal); Project administration (equal). **Mahmoud Aminlari:** Supervision (equal); Writing‐review & editing (equal).

## ETHICAL APPROVAL

This article does not contain any studies with human participants or animals performed by any of the authors.

## Data Availability

The data that support the findings of this study are available from the corresponding author upon reasonable request.
